# Propane-1,3-diammonium bis­[aqua­chlorido(4-hydroxy­pyridine-2,6-di­carboxyl­ato-κ^3^ *O* ^2^,*N*,*O* ^6^)mercurate(II)] tetra­hydrate

**DOI:** 10.1107/S1600536808022897

**Published:** 2008-07-26

**Authors:** Hossein Aghabozorg, Sara Bagheri, Mohammad Heidari, Mohammad Ghadermazi, Jafar Attar Gharamaleki

**Affiliations:** a Faculty of Chemistry, Tarbiat Moallem University, 49 Mofateh Avenue, Tehran, Iran; b Department of Chemistry, Islamic Azad University, North Tehran Branch, Tehran, Iran; c Department of Chemistry, Faculty of Science, University of Kurdistan, Sanandaj, Iran

## Abstract

The reaction of mercury(II) chloride dihydrate, propane-1,3-diamine and 4-hydroxy­pyridine-2,6-dicarboxylic acid in a 1:1:1 molar ratio in aqueous solution, resulted in the formation of the title compound, (C_3_H_12_N_2_)[Hg(C_7_H_3_NO_5_)Cl(H_2_O)]_2_·4H_2_O or (pnH_2_)[Hg(hypydc)Cl(H_2_O)]_2_·4H_2_O (where pn is propane-1,3-diamine and hypydcH_2_ is 4-hydroxy­pyridine-2,6-dicarboxylic acid). The metal atom is coordinated by one chloride group, one water mol­ecule *cis* to the chloride ligand and one (hypydc)^2−^ ligand. The coordinated water mol­ecule is almost perpendicular to the plane of the aromatic ring of (hypydc)^2−^. The geometry of the resulting HgClNO_3_ coordination can be described as distorted square-pyramidal. This structure also contains propane-1,3-diammonium (site symmetry 2) as a counter-ion and four uncoordinated water mol­ecules. There is a wide range of non-covalent inter­actions consisting of hydrogen bonding [of the types O—H⋯O, N—H⋯O and C—H⋯O, with *D*⋯*A* ranging from 2.548 (5) to 3.393 (6) Å] and ion pairing.

## Related literature

For related literature, see: Aghabozorg *et al.* (2007 ▶, 2008 ▶); Aghabozorg, Ghadermazi & Attar Gharamaleki (2006 ▶); Aghabozorg, Ghadermazi & Ramezanipour (2006 ▶); Agha­bozorg, Ghasemikhah *et al.* (2006 ▶); Ramezanipour *et al.* (2005 ▶).
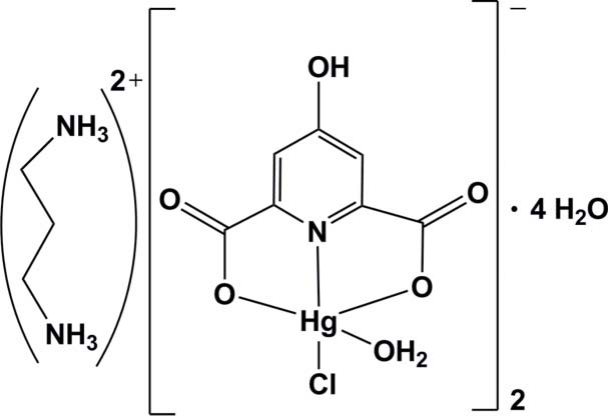


## Experimental

### 

#### Crystal data

(C_3_H_12_N_2_)[Hg(C_7_H_3_NO_5_)Cl(H_2_O)]_2_·4H_2_O
*M* *_r_* = 1018.53
Monoclinic, 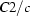

*a* = 29.2207 (13) Å
*b* = 6.7630 (3) Å
*c* = 15.4913 (7) Å
β = 114.5130 (10)°
*V* = 2785.5 (2) Å^3^
*Z* = 4
Mo *K*α radiation
μ = 11.28 mm^−1^
*T* = 100 (2) K
0.11 × 0.08 × 0.07 mm

#### Data collection

Bruker SMART APEXII diffractometer
Absorption correction: multi-scan (*SADABS*; Sheldrick, 1996 ▶) *T*_min_ = 0.284, *T*_max_ = 0.457
9362 measured reflections
3041 independent reflections
2632 reflections with *I* > 2σ(*I*)
*R*_int_ = 0.036

#### Refinement

*R*[*F* ^2^ > 2σ(*F* ^2^)] = 0.022
*wR*(*F* ^2^) = 0.047
*S* = 0.99
3041 reflections
187 parameters
H-atom parameters constrained
Δρ_max_ = 0.79 e Å^−3^
Δρ_min_ = −0.84 e Å^−3^

### 

Data collection: *APEX2* (Bruker, 2007 ▶); cell refinement: *APEX2*; data reduction: *APEX2*; program(s) used to solve structure: *SHELXTL* (Sheldrick, 2008 ▶); program(s) used to refine structure: *SHELXTL*; molecular graphics: *SHELXTL*; software used to prepare material for publication: *SHELXTL*.

## Supplementary Material

Crystal structure: contains datablocks I, global. DOI: 10.1107/S1600536808022897/om2244sup1.cif

Structure factors: contains datablocks I. DOI: 10.1107/S1600536808022897/om2244Isup2.hkl

Additional supplementary materials:  crystallographic information; 3D view; checkCIF report

## Figures and Tables

**Table 1:** Hydrogen-bond geometry (Å, °)

*D*—H⋯*A*	*D*—H	H⋯*A*	*D*⋯*A*	*D*—H⋯*A*
O3—H3⋯O4^i^	0.92	1.63	2.548 (5)	173
N2—H1*C*⋯O3*W*^ii^	0.89	2.02	2.830 (5)	150
N2—H1*D*⋯O2*W*^iii^	0.89	2.30	3.096 (6)	149
N2—H1*E*⋯O2*W*^iv^	0.89	1.96	2.824 (6)	165
O1*W*—H1*A*⋯O5^ii^	0.82	2.08	2.854 (5)	157
O1*W*—H1*B*⋯O2^v^	0.82	2.06	2.837 (6)	157
O2*W*—H2*B*⋯O1	0.85	1.98	2.771 (6)	154
O2*W*—H2*C*⋯O2^vi^	0.85	1.94	2.777 (5)	169
O3*W*—H3*A*⋯O3^v^	0.85	2.30	3.019 (6)	142
O3*W*—H3*B*⋯O5	0.85	1.93	2.766 (6)	169
C8—H8*B*⋯O1	0.97	2.45	3.393 (6)	163

Symmetry codes: (i) 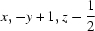; (ii) 
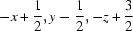
; (iii) 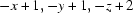; (iv) 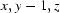; (v) 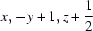; (vi) 
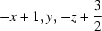
.

**Fig. 1.**
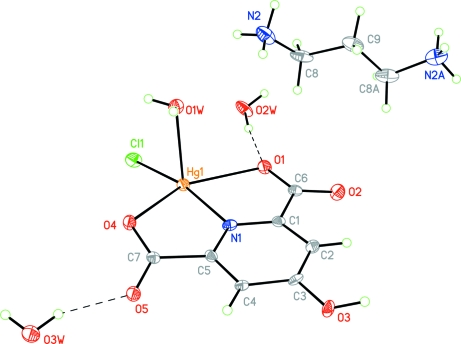
The molecular structure of the title compound, displacement ellipsoids are drawn at the 50% probability level. Hydrogen bonds are shown as dashed lines. Symmetry code A: -x + 1, y, -z + 3/2.

**Fig. 2.**
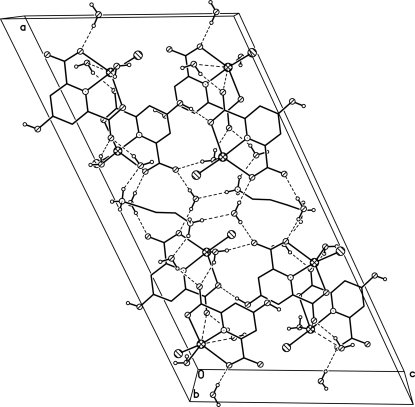
The crystal packing of the title compound viewed down the b axis, hydrogen bonds are shown as dashed lines.

## supplementary crystallographic information

### Comment

Recently, we have defined a plan to prepare water soluble proton-transfer compounds as novel self assembled systems that can function as suitable ligands in the synthesis of metal complexes. In this regard, we have reported cases in which protons transfer from pyridine-2,6-dicarboxylic acid, pydcH_2_, and benzene-1,2,4,5-tetracarboxylicacid, btcH_4_, to propane-1,3-diamine (pn) and 1,10-phenanthroline, (phen). These resulted in the formation of some novel proton transfer compounds such as (pnH_2_)(pydc).(pydcH_2_).2.5H_2_O (Aghabozorg, Ghadermazi, Ramezanipour, 2006), (pnH_2_)_2_(btc).2H_2_O (Aghabozorg, *et al.*, 2007) and (phenH)_4_(btcH_3_)_2_(btcH_2_) (Aghabozorg, Ghadermazi, Attar Gharamaleki, 2006). For more details and related literature see our recent review article (Aghabozorg, *et al.*, 2008).

The molecular structure and crystal packing diagram of the title compound are presented in Figs. 1 and 2, respectively.

The Hg^II^ atom is five-coordinated by one chloro group, one water molecule and one 4-hydroxypyridine-2,6-dicarboxylate, or (hypydc)^2–^, group which is coordinated through one pyridine N atom and two carboxylate O atoms. These distances are in good agreement with our two recently reported Hg^II^ structures (Aghabozorg, Ghasemikhah, Ghadermazi, *et al.*, 2006; Ramezanipour *et al.*, 2005).

The sum of the Cl1—Hg1—O1, O1—Hg1—N1, N1—Hg1—O4 and O4—Hg1—Cl1 bond angles equals 361.33 °, which indicates that these four atoms are almost located in the plane. As it can be seen, the O1W atom of the coordinated water molecule occupies the axial position, while the O1, O4, N1 and Cl1 atoms form the equatorial plane of the square pyramid. The O1W—Hg1—Cl1, O1W—Hg1—N1, O1W—Hg1—O1 and O1W—Hg1—O4 angles are 94.63 (7), 96.83 (11), 91.72 (9) and 82.81 (9)^°^, respectively, indicating that the coordinated water molecule is located at *cis* position to the chloro ligand and is also almost perpendicular to the square plane of the pyramid. Therefore, the geometry of the resulting HgClNO_3_ coordination can be described as distorted square pyramidal. The molecular structure of the title compound also contains propane-1,3-diammonium (site symmetry 2) as counter-ion and four uncoordinated water molecules. In the crystal structure, there is a wide range of non-covalent interactions consisting of hydrogen bonding (of the type O—H···O, N—H···O and C—H···O with D···A ranging from 2.548 (5) Å to 3.393 (6) Å) and ion pairing (Table 1).

### Experimental

Aqueous solutions of HgCl_2_.2H_2_O (76 mg, 0.2 mmol), propane-1,3-diamine (18 mg, 0.2 mmol) and 4-hydroxypyridine-2,6-dicarboxylic acid (72 mg, 0.2 mmol) were mixed in a 1:1:1 molar ratio, and the reaction mixture was heated at about 313 K for 2 h. Colourless crystals of the title compound were obtained from the solution after three weeks at room temperature.

### Refinement

The hydrogen atoms of the NH_3_ and OH groups, and also H atoms of water molecules were found in difference Fourier synthesis. The H(C) atom positions were calculated. All H(N) and H(O) atoms were refined in isotropic approximation in rigid model, the H(C) atoms were refined in isotropic approximation in riding model with with the *U*_iso_(H) parameters equal to 1.2 *U*_eq_(Ci) and 1.5 *U*_eq_(Cii) for OH, NH3 group and water molecules, where U(C) are the equivalent thermal parameters of the atoms to which corresponding H atoms are bonded.

### Crystal data

**Table:**

(C_3_H_12_N_2_)[Hg(C_7_H_3_NO_5_)Cl(H_2_O)]_2_·4H_2_O	*F*_000_ = 1928
*M**_r_* = 1018.53	*D*_x_ = 2.429 Mg m^−^^3^
Monoclinic, *C*2/*c*	Mo *K*α radiation λ = 0.71073 Å
*a* = 29.2207 (13) Å	Cell parameters from 2868 reflections
*b* = 6.7630 (3) Å	θ = 3–27º
*c* = 15.4913 (7) Å	µ = 11.28 mm^−^^1^
β = 114.5130 (10)º	*T* = 100 (2) K
*V* = 2785.5 (2) Å^3^	Prism, colourless
*Z* = 4	0.11 × 0.08 × 0.07 mm

### Data collection

**Table:**

Bruker SMART APEXII diffractometer	3041 independent reflections
Radiation source: fine-focus sealed tube	2632 reflections with *I* > 2σ(*I*)
Monochromator: graphite	*R*_int_ = 0.036
*T* = 100(2) K	θ_max_ = 27.0º
φ and ω scans	θ_min_ = 1.5º
Absorption correction: multi-scan(SADABS; Sheldrick, 1996)	*h* = −37→36
*T*_min_ = 0.284, *T*_max_ = 0.457	*k* = −8→8
9362 measured reflections	*l* = −19→19

### Refinement

**Table:**

Refinement on *F*^2^	Secondary atom site location: difference Fourier map
Least-squares matrix: full	Hydrogen site location: mixed
*R*[*F*^2^ > 2σ(*F*^2^)] = 0.022	H-atom parameters constrained
*wR*(*F*^2^) = 0.047	*w* = 1/[σ^2^(*F*_o_^2^) + (0.02*P*)^2^] where *P* = (*F*_o_^2^ + 2*F*_c_^2^)/3
*S* = 0.99	(Δ/σ)_max_ = 0.003
3041 reflections	Δρ_max_ = 0.79 e Å^−^^3^
187 parameters	Δρ_min_ = −0.84 e Å^−^^3^
Primary atom site location: structure-invariant direct methods	Extinction correction: none

### Special details

**Table:**

Geometry. All e.s.d.'s (except the e.s.d. in the dihedral angle between two l.s. planes) are estimated using the full covariance matrix. The cell e.s.d.'s are taken into account individually in the estimation of e.s.d.'s in distances, angles and torsion angles; correlations between e.s.d.'s in cell parameters are only used when they are defined by crystal symmetry. An approximate (isotropic) treatment of cell e.s.d.'s is used for estimating e.s.d.'s involving l.s. planes.
Refinement. Refinement of *F*^2^ against ALL reflections. The weighted *R*-factor *wR* and goodness of fit *S* are based on *F*^2^, conventional *R*-factors *R* are based on *F*, with *F* set to zero for negative *F*^2^. The threshold expression of *F*^2^ > σ(*F*^2^) is used only for calculating *R*-factors(gt) *etc*. and is not relevant to the choice of reflections for refinement. *R*-factors based on *F*^2^ are statistically about twice as large as those based on *F*, and *R*- factors based on ALL data will be even larger.

### Fractional atomic coordinates and isotropic or equivalent isotropic displacement parameters (Å^2^)

**Table:**

	*x*	*y*	*z*	*U*_iso_*/*U*_eq_	
Hg1	0.360437 (6)	0.72342 (2)	0.843452 (11)	0.01284 (6)	
Cl1	0.40073 (4)	0.88117 (16)	0.98816 (7)	0.0205 (2)	
O1	0.41510 (10)	0.6883 (4)	0.7583 (2)	0.0163 (6)	
O2	0.40827 (11)	0.6472 (5)	0.6102 (2)	0.0200 (7)	
O3	0.22507 (10)	0.4439 (4)	0.43123 (19)	0.0161 (6)	
H3	0.2422	0.4065	0.3956	0.024*	
O4	0.27295 (10)	0.6300 (4)	0.8299 (2)	0.0159 (6)	
O5	0.19566 (10)	0.6269 (5)	0.7138 (2)	0.0173 (6)	
N1	0.31418 (12)	0.6356 (5)	0.7000 (2)	0.0115 (7)	
C1	0.33511 (15)	0.6083 (6)	0.6378 (3)	0.0118 (8)	
C2	0.30679 (15)	0.5421 (6)	0.5469 (3)	0.0135 (9)	
H2A	0.3218	0.5200	0.5053	0.016*	
C3	0.25554 (15)	0.5084 (6)	0.5177 (3)	0.0118 (9)	
C4	0.23390 (15)	0.5464 (6)	0.5813 (3)	0.0120 (8)	
H4A	0.1994	0.5324	0.5627	0.014*	
C5	0.26445 (15)	0.6044 (6)	0.6713 (3)	0.0113 (8)	
C6	0.39097 (15)	0.6514 (6)	0.6713 (3)	0.0130 (8)	
C7	0.24210 (15)	0.6239 (6)	0.7438 (3)	0.0106 (8)	
N2	0.47091 (14)	0.1672 (6)	0.8789 (3)	0.0266 (9)	
H1C	0.4384	0.1443	0.8438	0.040*	
H1D	0.4744	0.2326	0.9311	0.040*	
H1E	0.4873	0.0528	0.8949	0.040*	
C8	0.49190 (17)	0.2877 (7)	0.8234 (3)	0.0254 (11)	
H8A	0.5237	0.3455	0.8659	0.030*	
H8B	0.4689	0.3945	0.7916	0.030*	
C9	0.5000	0.1595 (9)	0.7500	0.0249 (15)	
H9A	0.4711	0.0763	0.7190	0.030*	
O1W	0.38232 (10)	0.3754 (4)	0.91250 (19)	0.0169 (6)	
H1A	0.3582	0.3001	0.8895	0.025*	
H1B	0.3848	0.3976	0.9663	0.025*	
O2W	0.50594 (11)	0.7731 (4)	0.9089 (2)	0.0245 (7)	
H2B	0.4842	0.7451	0.8535	0.037*	
H2C	0.5315	0.7479	0.8977	0.037*	
O3W	0.12234 (11)	0.6629 (5)	0.7822 (2)	0.0245 (7)	
H3A	0.1428	0.6436	0.8395	0.037*	
H3B	0.1438	0.6364	0.7600	0.037*	

### Atomic displacement parameters (Å^2^)

**Table:**

	*U*^11^	*U*^22^	*U*^33^	*U*^12^	*U*^13^	*U*^23^
Hg1	0.00977 (9)	0.01538 (9)	0.01123 (8)	−0.00051 (7)	0.00222 (6)	−0.00185 (7)
Cl1	0.0158 (5)	0.0230 (6)	0.0160 (5)	0.0004 (4)	−0.0002 (4)	−0.0066 (4)
O1	0.0102 (15)	0.0221 (16)	0.0141 (15)	−0.0007 (12)	0.0025 (12)	−0.0031 (13)
O2	0.0127 (16)	0.0302 (17)	0.0187 (16)	−0.0032 (13)	0.0082 (13)	0.0011 (14)
O3	0.0106 (15)	0.0279 (17)	0.0084 (14)	−0.0034 (12)	0.0026 (12)	−0.0056 (12)
O4	0.0095 (15)	0.0257 (17)	0.0120 (15)	−0.0008 (12)	0.0038 (12)	−0.0012 (13)
O5	0.0103 (16)	0.0285 (17)	0.0128 (15)	0.0006 (13)	0.0044 (13)	−0.0003 (13)
N1	0.0085 (17)	0.0113 (16)	0.0103 (17)	−0.0020 (13)	−0.0004 (14)	−0.0009 (14)
C1	0.012 (2)	0.010 (2)	0.012 (2)	0.0019 (15)	0.0034 (17)	0.0010 (16)
C2	0.017 (2)	0.011 (2)	0.014 (2)	0.0006 (16)	0.0073 (18)	0.0031 (16)
C3	0.013 (2)	0.011 (2)	0.008 (2)	−0.0001 (16)	0.0018 (18)	0.0040 (15)
C4	0.008 (2)	0.015 (2)	0.009 (2)	0.0020 (16)	0.0006 (17)	0.0015 (16)
C5	0.010 (2)	0.011 (2)	0.014 (2)	0.0012 (15)	0.0068 (18)	0.0022 (16)
C6	0.012 (2)	0.0094 (19)	0.016 (2)	0.0002 (16)	0.0040 (18)	0.0009 (17)
C7	0.012 (2)	0.0068 (19)	0.012 (2)	0.0007 (15)	0.0039 (17)	−0.0015 (15)
N2	0.018 (2)	0.028 (2)	0.031 (2)	0.0039 (17)	0.0067 (18)	−0.0086 (18)
C8	0.013 (2)	0.017 (2)	0.033 (3)	0.0037 (18)	−0.004 (2)	−0.003 (2)
C9	0.013 (3)	0.017 (3)	0.040 (4)	0.000	0.005 (3)	0.000
O1W	0.0174 (17)	0.0173 (15)	0.0139 (15)	−0.0014 (12)	0.0046 (13)	−0.0020 (12)
O2W	0.0126 (16)	0.0283 (18)	0.0305 (18)	0.0014 (13)	0.0067 (14)	−0.0098 (15)
O3W	0.0126 (16)	0.039 (2)	0.0191 (16)	0.0034 (14)	0.0037 (14)	0.0031 (15)

### Geometric parameters (Å, °)

**Table:**

Hg1—N1	2.151 (3)	C2—H2A	0.9300
Hg1—Cl1	2.3151 (10)	C3—C4	1.397 (5)
Hg1—O1	2.469 (3)	C4—C5	1.365 (6)
Hg1—O1W	2.555 (3)	C4—Hg1^ii^	4.049 (4)
Hg1—O4	2.556 (3)	C4—H4A	0.9300
Hg1—C1	3.058 (4)	C5—C7	1.521 (5)
Hg1—C5	3.069 (4)	C7—Hg1^ii^	3.844 (4)
Hg1—O5^i^	3.117 (3)	N2—C8	1.489 (6)
Hg1—C6	3.182 (4)	N2—H1C	0.8900
Hg1—C7	3.219 (4)	N2—H1D	0.8900
Hg1—O3W^i^	3.700 (3)	N2—H1E	0.8900
Hg1—C7^i^	3.844 (4)	C8—C9	1.524 (6)
O1—C6	1.261 (5)	C8—H8A	0.9700
O2—C6	1.244 (5)	C8—H8B	0.9700
O3—C3	1.337 (5)	C9—C8^iii^	1.524 (6)
O3—H3	0.9220	C9—H9A	0.9601
O4—C7	1.263 (5)	O1W—H1A	0.8205
O5—C7	1.239 (5)	O1W—H1B	0.8199
O5—Hg1^ii^	3.117 (3)	O2W—H2B	0.8500
N1—C5	1.348 (5)	O2W—H2C	0.8499
N1—C1	1.351 (5)	O3W—Hg1^ii^	3.700 (3)
C1—C2	1.379 (6)	O3W—H3A	0.8501
C1—C6	1.522 (5)	O3W—H3B	0.8501
C2—C3	1.392 (6)		
			
N1—Hg1—Cl1	167.66 (9)	C3—O3—H3	112.8
N1—Hg1—O1	71.94 (11)	C7—O4—Hg1	110.2 (2)
Cl1—Hg1—O1	112.32 (7)	C7—O5—Hg1^ii^	117.2 (2)
N1—Hg1—O1W	96.83 (11)	C5—N1—C1	119.2 (3)
Cl1—Hg1—O1W	94.63 (7)	C5—N1—Hg1	120.9 (3)
O1—Hg1—O1W	91.72 (9)	C1—N1—Hg1	119.9 (3)
N1—Hg1—O4	70.64 (11)	N1—C1—C2	121.0 (4)
Cl1—Hg1—O4	106.43 (7)	N1—C1—C6	118.0 (3)
O1—Hg1—O4	141.18 (9)	C2—C1—C6	121.0 (4)
O1W—Hg1—O4	82.81 (9)	C2—C1—Hg1	158.4 (3)
N1—Hg1—C1	22.53 (11)	C6—C1—Hg1	80.5 (2)
Cl1—Hg1—C1	158.80 (8)	C1—C2—C3	119.6 (4)
O1—Hg1—C1	49.41 (10)	C1—C2—H2A	120.2
O1W—Hg1—C1	96.44 (10)	C3—C2—H2A	120.2
O4—Hg1—C1	92.87 (10)	O3—C3—C2	123.9 (4)
N1—Hg1—C5	22.16 (11)	O3—C3—C4	117.3 (4)
Cl1—Hg1—C5	151.22 (8)	C2—C3—C4	118.8 (4)
O1—Hg1—C5	94.09 (10)	C5—C4—C3	118.5 (4)
O1W—Hg1—C5	95.85 (10)	C5—C4—Hg1^ii^	95.5 (2)
O4—Hg1—C5	48.81 (9)	C3—C4—Hg1^ii^	131.7 (3)
C1—Hg1—C5	44.68 (10)	C5—C4—H4A	120.7
N1—Hg1—O5^i^	85.23 (10)	C3—C4—H4A	120.7
Cl1—Hg1—O5^i^	82.43 (6)	N1—C5—C4	122.7 (4)
O1—Hg1—O5^i^	108.22 (8)	N1—C5—C7	118.5 (3)
O1W—Hg1—O5^i^	159.50 (8)	C4—C5—C7	118.7 (3)
O4—Hg1—O5^i^	78.64 (8)	C4—C5—Hg1	159.7 (3)
C1—Hg1—O5^i^	93.15 (9)	C7—C5—Hg1	81.6 (2)
C5—Hg1—O5^i^	78.42 (9)	O2—C6—O1	126.5 (4)
N1—Hg1—C6	50.66 (11)	O2—C6—C1	116.9 (4)
Cl1—Hg1—C6	132.49 (8)	O1—C6—C1	116.6 (3)
O1—Hg1—C6	21.38 (10)	O2—C6—Hg1	169.9 (3)
O1W—Hg1—C6	95.82 (9)	O1—C6—Hg1	45.54 (19)
O4—Hg1—C6	120.80 (10)	C1—C6—Hg1	71.4 (2)
C1—Hg1—C6	28.14 (10)	O5—C7—O4	125.9 (4)
C5—Hg1—C6	72.81 (10)	O5—C7—C5	117.6 (3)
O5^i^—Hg1—C6	101.11 (9)	O4—C7—C5	116.5 (3)
N1—Hg1—C7	50.00 (11)	O5—C7—Hg1	165.4 (3)
Cl1—Hg1—C7	125.05 (7)	O4—C7—Hg1	48.15 (19)
O1—Hg1—C7	121.87 (9)	C5—C7—Hg1	70.6 (2)
O1W—Hg1—C7	92.32 (9)	O5—C7—Hg1^ii^	46.1 (2)
O4—Hg1—C7	21.60 (9)	O4—C7—Hg1^ii^	120.3 (3)
C1—Hg1—C7	72.52 (10)	C5—C7—Hg1^ii^	101.0 (2)
C5—Hg1—C7	27.86 (9)	Hg1—C7—Hg1^ii^	147.28 (12)
O5^i^—Hg1—C7	73.33 (9)	C8—N2—H1C	109.5
C6—Hg1—C7	100.66 (10)	C8—N2—H1D	109.5
N1—Hg1—O3W^i^	80.95 (10)	H1C—N2—H1D	109.5
Cl1—Hg1—O3W^i^	90.78 (6)	C8—N2—H1E	109.5
O1—Hg1—O3W^i^	62.42 (8)	H1C—N2—H1E	109.5
O1W—Hg1—O3W^i^	153.57 (8)	H1D—N2—H1E	109.5
O4—Hg1—O3W^i^	120.33 (8)	N2—C8—C9	110.3 (4)
C1—Hg1—O3W^i^	71.54 (9)	N2—C8—H8A	109.6
C5—Hg1—O3W^i^	91.63 (9)	C9—C8—H8A	109.6
O5^i^—Hg1—O3W^i^	46.93 (7)	N2—C8—H8B	109.6
C6—Hg1—O3W^i^	62.32 (9)	C9—C8—H8B	109.6
C7—Hg1—O3W^i^	105.62 (8)	H8A—C8—H8B	108.1
N1—Hg1—C7^i^	76.55 (11)	C8—C9—C8^iii^	110.6 (5)
Cl1—Hg1—C7^i^	91.42 (6)	C8—C9—H9A	109.3
O1—Hg1—C7^i^	117.56 (9)	C8^iii^—C9—H9A	109.6
O1W—Hg1—C7^i^	145.03 (9)	Hg1—O1W—H1A	111.7
O4—Hg1—C7^i^	62.49 (9)	Hg1—O1W—H1B	99.1
C1—Hg1—C7^i^	89.81 (9)	H1A—O1W—H1B	104.6
C5—Hg1—C7^i^	65.25 (9)	Hg1—O2W—H2B	56.6
O5^i^—Hg1—C7^i^	16.66 (8)	Hg1—O2W—H2C	150.6
C6—Hg1—C7^i^	105.18 (9)	H2B—O2W—H2C	95.7
C7—Hg1—C7^i^	56.86 (4)	Hg1^ii^—O3W—H3A	103.6
O3W^i^—Hg1—C7^i^	60.36 (7)	Hg1^ii^—O3W—H3B	48.2
C6—O1—Hg1	113.1 (2)	H3A—O3W—H3B	94.0
			
N1—Hg1—O1—C6	5.0 (3)	C7—Hg1—C5—C4	−175.8 (9)
Cl1—Hg1—O1—C6	−162.6 (3)	O3W^i^—Hg1—C5—C4	62.7 (8)
O1W—Hg1—O1—C6	101.6 (3)	C7^i^—Hg1—C5—C4	118.8 (8)
O4—Hg1—O1—C6	21.0 (3)	N1—Hg1—C5—C7	177.6 (4)
C1—Hg1—O1—C6	4.6 (3)	Cl1—Hg1—C5—C7	−26.9 (3)
C5—Hg1—O1—C6	5.7 (3)	O1—Hg1—C5—C7	176.0 (2)
O5^i^—Hg1—O1—C6	−73.5 (3)	O1W—Hg1—C5—C7	83.9 (2)
C7—Hg1—O1—C6	7.8 (3)	O4—Hg1—C5—C7	8.77 (19)
O3W^i^—Hg1—O1—C6	−84.1 (3)	C1—Hg1—C5—C7	177.1 (3)
C7^i^—Hg1—O1—C6	−58.5 (3)	O5^i^—Hg1—C5—C7	−76.2 (2)
N1—Hg1—O4—C7	−15.6 (3)	C6—Hg1—C5—C7	178.2 (2)
Cl1—Hg1—O4—C7	151.8 (2)	O3W^i^—Hg1—C5—C7	−121.5 (2)
O1—Hg1—O4—C7	−31.7 (3)	C7^i^—Hg1—C5—C7	−65.42 (19)
O1W—Hg1—O4—C7	−115.5 (3)	Hg1—O1—C6—O2	171.9 (3)
C1—Hg1—O4—C7	−19.3 (3)	Hg1—O1—C6—C1	−7.9 (4)
C5—Hg1—O4—C7	−11.2 (2)	N1—C1—C6—O2	−172.1 (4)
O5^i^—Hg1—O4—C7	73.3 (3)	C2—C1—C6—O2	8.4 (6)
C6—Hg1—O4—C7	−22.9 (3)	Hg1—C1—C6—O2	−173.9 (4)
O3W^i^—Hg1—O4—C7	50.9 (3)	N1—C1—C6—O1	7.8 (5)
C7^i^—Hg1—O4—C7	69.0 (2)	C2—C1—C6—O1	−171.7 (4)
Cl1—Hg1—N1—C5	−69.5 (6)	Hg1—C1—C6—O1	6.0 (3)
O1—Hg1—N1—C5	178.3 (3)	N1—C1—C6—Hg1	1.8 (3)
O1W—Hg1—N1—C5	88.7 (3)	C2—C1—C6—Hg1	−177.7 (4)
O4—Hg1—N1—C5	8.9 (3)	N1—Hg1—C6—O2	145.7 (18)
C1—Hg1—N1—C5	179.1 (5)	Cl1—Hg1—C6—O2	−18.5 (18)
O5^i^—Hg1—N1—C5	−70.8 (3)	O1—Hg1—C6—O2	−40.5 (16)
C6—Hg1—N1—C5	−179.3 (4)	O1W—Hg1—C6—O2	−120.2 (17)
C7—Hg1—N1—C5	1.4 (3)	O4—Hg1—C6—O2	154.7 (17)
O3W^i^—Hg1—N1—C5	−117.9 (3)	C1—Hg1—C6—O2	147.0 (18)
C7^i^—Hg1—N1—C5	−56.4 (3)	C5—Hg1—C6—O2	145.4 (17)
Cl1—Hg1—N1—C1	111.5 (4)	O5^i^—Hg1—C6—O2	71.4 (17)
O1—Hg1—N1—C1	−0.8 (3)	C7—Hg1—C6—O2	146.3 (17)
O1W—Hg1—N1—C1	−90.3 (3)	O3W^i^—Hg1—C6—O2	44.1 (17)
O4—Hg1—N1—C1	−170.2 (3)	C7^i^—Hg1—C6—O2	88.0 (17)
C5—Hg1—N1—C1	−179.1 (5)	N1—Hg1—C6—O1	−173.8 (3)
O5^i^—Hg1—N1—C1	110.2 (3)	Cl1—Hg1—C6—O1	22.0 (3)
C6—Hg1—N1—C1	1.6 (3)	O1W—Hg1—C6—O1	−79.8 (3)
C7—Hg1—N1—C1	−177.6 (4)	O4—Hg1—C6—O1	−164.8 (2)
O3W^i^—Hg1—N1—C1	63.1 (3)	C1—Hg1—C6—O1	−172.5 (4)
C7^i^—Hg1—N1—C1	124.6 (3)	C5—Hg1—C6—O1	−174.1 (3)
C5—N1—C1—C2	−2.5 (6)	O5^i^—Hg1—C6—O1	111.9 (3)
Hg1—N1—C1—C2	176.6 (3)	C7—Hg1—C6—O1	−173.2 (3)
C5—N1—C1—C6	178.0 (3)	O3W^i^—Hg1—C6—O1	84.6 (3)
Hg1—N1—C1—C6	−2.9 (5)	C7^i^—Hg1—C6—O1	128.5 (3)
C5—N1—C1—Hg1	−179.1 (5)	N1—Hg1—C6—C1	−1.3 (2)
Cl1—Hg1—C1—N1	−146.6 (3)	Cl1—Hg1—C6—C1	−165.48 (17)
O1—Hg1—C1—N1	179.0 (3)	O1—Hg1—C6—C1	172.5 (4)
O1W—Hg1—C1—N1	92.3 (3)	O1W—Hg1—C6—C1	92.8 (2)
O4—Hg1—C1—N1	9.2 (3)	O4—Hg1—C6—C1	7.7 (2)
C5—Hg1—C1—N1	0.5 (3)	C5—Hg1—C6—C1	−1.6 (2)
O5^i^—Hg1—C1—N1	−69.5 (3)	O5^i^—Hg1—C6—C1	−75.6 (2)
C6—Hg1—C1—N1	−177.4 (4)	C7—Hg1—C6—C1	−0.7 (2)
C7—Hg1—C1—N1	1.9 (3)	O3W^i^—Hg1—C6—C1	−102.9 (2)
O3W^i^—Hg1—C1—N1	−111.8 (3)	C7^i^—Hg1—C6—C1	−59.0 (2)
C7^i^—Hg1—C1—N1	−53.2 (3)	Hg1^ii^—O5—C7—O4	−99.8 (4)
N1—Hg1—C1—C2	−8.0 (7)	Hg1^ii^—O5—C7—C5	78.1 (4)
Cl1—Hg1—C1—C2	−154.6 (6)	Hg1^ii^—O5—C7—Hg1	−160.3 (10)
O1—Hg1—C1—C2	171.0 (8)	Hg1—O4—C7—O5	−162.9 (3)
O1W—Hg1—C1—C2	84.3 (8)	Hg1—O4—C7—C5	19.2 (4)
O4—Hg1—C1—C2	1.2 (8)	Hg1—O4—C7—Hg1^ii^	141.68 (15)
C5—Hg1—C1—C2	−7.5 (7)	N1—C5—C7—O5	168.5 (4)
O5^i^—Hg1—C1—C2	−77.5 (8)	C4—C5—C7—O5	−14.8 (5)
C6—Hg1—C1—C2	174.6 (9)	Hg1—C5—C7—O5	166.9 (3)
C7—Hg1—C1—C2	−6.1 (7)	N1—C5—C7—O4	−13.4 (5)
O3W^i^—Hg1—C1—C2	−119.9 (8)	C4—C5—C7—O4	163.3 (4)
C7^i^—Hg1—C1—C2	−61.2 (8)	Hg1—C5—C7—O4	−15.0 (3)
N1—Hg1—C1—C6	177.4 (4)	N1—C5—C7—Hg1	1.6 (3)
Cl1—Hg1—C1—C6	30.7 (4)	C4—C5—C7—Hg1	178.3 (4)
O1—Hg1—C1—C6	−3.58 (19)	N1—C5—C7—Hg1^ii^	−145.6 (3)
O1W—Hg1—C1—C6	−90.3 (2)	C4—C5—C7—Hg1^ii^	31.2 (4)
O4—Hg1—C1—C6	−173.4 (2)	Hg1—C5—C7—Hg1^ii^	−147.18 (11)
C5—Hg1—C1—C6	177.9 (3)	N1—Hg1—C7—O5	−128.0 (12)
O5^i^—Hg1—C1—C6	107.8 (2)	Cl1—Hg1—C7—O5	37.8 (12)
C7—Hg1—C1—C6	179.3 (2)	O1—Hg1—C7—O5	−131.5 (11)
O3W^i^—Hg1—C1—C6	65.5 (2)	O1W—Hg1—C7—O5	135.1 (11)
C7^i^—Hg1—C1—C6	124.2 (2)	O4—Hg1—C7—O5	71.4 (11)
N1—C1—C2—C3	2.0 (6)	C1—Hg1—C7—O5	−128.9 (11)
C6—C1—C2—C3	−178.5 (4)	C5—Hg1—C7—O5	−126.8 (12)
Hg1—C1—C2—C3	7.7 (10)	O5^i^—Hg1—C7—O5	−30.0 (11)
C1—C2—C3—O3	−179.8 (4)	C6—Hg1—C7—O5	−128.6 (11)
C1—C2—C3—C4	1.1 (6)	O3W^i^—Hg1—C7—O5	−64.6 (11)
O3—C3—C4—C5	177.3 (4)	C7^i^—Hg1—C7—O5	−27.3 (10)
C2—C3—C4—C5	−3.6 (6)	N1—Hg1—C7—O4	160.7 (3)
O3—C3—C4—Hg1^ii^	48.2 (5)	Cl1—Hg1—C7—O4	−33.6 (3)
C2—C3—C4—Hg1^ii^	−132.7 (3)	O1—Hg1—C7—O4	157.2 (2)
C1—N1—C5—C4	−0.2 (6)	O1W—Hg1—C7—O4	63.7 (3)
Hg1—N1—C5—C4	−179.2 (3)	C1—Hg1—C7—O4	159.7 (3)
C1—N1—C5—C7	176.4 (3)	C5—Hg1—C7—O4	161.8 (4)
Hg1—N1—C5—C7	−2.7 (5)	O5^i^—Hg1—C7—O4	−101.4 (3)
C1—N1—C5—Hg1	179.1 (5)	C6—Hg1—C7—O4	160.1 (3)
C3—C4—C5—N1	3.2 (6)	O3W^i^—Hg1—C7—O4	−135.9 (2)
Hg1^ii^—C4—C5—N1	147.6 (3)	C7^i^—Hg1—C7—O4	−98.7 (3)
C3—C4—C5—C7	−173.3 (3)	N1—Hg1—C7—C5	−1.2 (2)
Hg1^ii^—C4—C5—C7	−29.0 (4)	Cl1—Hg1—C7—C5	164.56 (18)
C3—C4—C5—Hg1	1.9 (10)	O1—Hg1—C7—C5	−4.7 (2)
Hg1^ii^—C4—C5—Hg1	146.3 (7)	O1W—Hg1—C7—C5	−98.1 (2)
Cl1—Hg1—C5—N1	155.4 (3)	O4—Hg1—C7—C5	−161.8 (4)
O1—Hg1—C5—N1	−1.6 (3)	C1—Hg1—C7—C5	−2.1 (2)
O1W—Hg1—C5—N1	−93.8 (3)	O5^i^—Hg1—C7—C5	96.8 (2)
O4—Hg1—C5—N1	−168.9 (3)	C6—Hg1—C7—C5	−1.8 (2)
C1—Hg1—C5—N1	−0.5 (3)	O3W^i^—Hg1—C7—C5	62.2 (2)
O5^i^—Hg1—C5—N1	106.2 (3)	C7^i^—Hg1—C7—C5	99.5 (2)
C6—Hg1—C5—N1	0.5 (3)	N1—Hg1—C7—Hg1^ii^	78.7 (2)
C7—Hg1—C5—N1	−177.6 (4)	Cl1—Hg1—C7—Hg1^ii^	−115.6 (2)
O3W^i^—Hg1—C5—N1	60.9 (3)	O1—Hg1—C7—Hg1^ii^	75.2 (2)
C7^i^—Hg1—C5—N1	116.9 (3)	O1W—Hg1—C7—Hg1^ii^	−18.3 (2)
N1—Hg1—C5—C4	1.9 (7)	O4—Hg1—C7—Hg1^ii^	−82.0 (3)
Cl1—Hg1—C5—C4	157.3 (7)	C1—Hg1—C7—Hg1^ii^	77.8 (2)
O1—Hg1—C5—C4	0.2 (8)	C5—Hg1—C7—Hg1^ii^	79.9 (3)
O1W—Hg1—C5—C4	−91.9 (8)	O5^i^—Hg1—C7—Hg1^ii^	176.6 (2)
O4—Hg1—C5—C4	−167.0 (9)	C6—Hg1—C7—Hg1^ii^	78.1 (2)
C1—Hg1—C5—C4	1.3 (8)	O3W^i^—Hg1—C7—Hg1^ii^	142.1 (2)
O5^i^—Hg1—C5—C4	108.0 (8)	C7^i^—Hg1—C7—Hg1^ii^	179.4 (3)
C6—Hg1—C5—C4	2.4 (8)	N2—C8—C9—C8^iii^	165.1 (4)

Symmetry codes: (i) −*x*+1/2, *y*+1/2, −*z*+3/2; (ii) −*x*+1/2, *y*−1/2, −*z*+3/2; (iii) −*x*+1, *y*, −*z*+3/2.

### Hydrogen-bond geometry (Å, °)

**Table:**

*D*—H···*A*	*D*—H	H···*A*	*D*···*A*	*D*—H···*A*
O3—H3···O4^iv^	0.92	1.63	2.548 (5)	173
N2—H1C···O3W^ii^	0.89	2.03	2.830 (5)	150
N2—H1D···O2W^v^	0.89	2.30	3.096 (6)	149
N2—H1E···O2W^vi^	0.89	1.96	2.824 (6)	165
O1W—H1A···O5^ii^	0.82	2.08	2.854 (5)	157
O1W—H1B···O2^vii^	0.82	2.07	2.837 (6)	157
O2W—H2B···O1	0.85	1.98	2.771 (6)	154
O2W—H2C···O2^iii^	0.85	1.94	2.777 (5)	169
O3W—H3A···O3^vii^	0.85	2.30	3.019 (6)	142
O3W—H3B···O5	0.85	1.93	2.766 (6)	169
C8—H8B···O1	0.97	2.45	3.393 (6)	163

Symmetry codes: (iv) *x*, −*y*+1, *z*−1/2; (ii) −*x*+1/2, *y*−1/2, −*z*+3/2; (v) −*x*+1, −*y*+1, −*z*+2; (vi) *x*, *y*−1, *z*; (vii) *x*, −*y*+1, *z*+1/2;  (iii) −*x*+1, *y*, −*z*+3/2.
